# Excision of the Astragalus and Os Calcis, with Some General Observations on the Excision of Joints

**Published:** 1851-05

**Authors:** Thomas Wakley


*Excision of the Astragalus and Os Calcis, with some general observations on the Excision of Joints.* By Mr. Thomas Wakley.—Excision of the extremities of the bones of joints, was first practised in 1782, by Mr. Park, one of the surgeons of the Liverpool Infirmary. In the same year, Moreau excised the extremities of the bones of the elbow joint, and in a fortnight after the operation the patient was so well, that he was allowed to go wherever he pleased, with his arm supported in a case. It was at first powerless, but it slowly regained its strength, and the man could ultimately thrash corn and hold the plough with it. In the same year, (1782,) the excision of the elbow-joint was twice performed by another French surgeon, and in both cases with complete success, the patients retaining considerable movement of the arms. Operations for the removal of the extremities of bones entering into the formation of the different joints, have been practiced since by English as well as continental surgeons; and the general success which has attended them fully justify their repetition. The limbs which have been saved are, in many instances, living monuments of the triumphs of surgery. Until the last few years, the ankle-joint was not considered favorable for the operation of excision, its complicated machinery and anatomical relations being considered impediments to the complete excision of the ends of the bones.

Some surgeons still adhere to the opinion, that it is necessary to amputate the foot for disease of the bones of the tarsus; thus we have the operations of Chopart and Syme for the removal of portions of the foot. However, there are cases on record, and there is evidence afforded by the condition of patients themselves, proving in some cases the superiority of excision of the ankle-joint over amputation of the foot. While contemplating the operation, an account of which formed the principal feature in his paper, Mr. Wakley received great encouragement from reading the cases of compound injuries to the ankle-joint, narrated by Sir Astley Cooper in his treatise on Dislocations and Fractures, as it was shown by this celebrated surgeon that these cases had recovered and done well after the most severe forms of dislocation and fracture, where even the astragalus was dislodged or extracted. Thus Mr. Wakley had operated three times upon the ankle-joint, in two cases partially excising the bones of the joint. In both cases amputation had been considered necessary; but the modified operation had saved the limbs. The third case was that of William Brown, lately exhibited to the Society as having undergone excision of the astragalus and calcis. The operation, which was perfectly successful, but the steps of which it is unnecessary to relate, was of a novel character—the astragalus and calcaneum, together with the malleolar processes of the tibia and fibula, being removed for extensive disease existing in the two first-named bones.—*Medical Times, April* 12, 1851.

